# PHYSICAL, COGNITIVE AND MENTAL CHARACTERISTICS AND DIGITAL HEALTH TECHNOLOGY USE AMONG PREFRAIL OLDER ADULTS

**DOI:** 10.1093/geroni/igae098.4045

**Published:** 2024-12-31

**Authors:** Takahisa Ohta, Sho Hatanaka, Takashi Shida, Narumi Kojima, Yosuke Osuka, Tsuyoshi Okamura, Hirohiko Hirano, Shuichi Awata

**Affiliations:** Tokyo Metropolitan Institute for Geriatrics and Gerontology, Itabashi, Tokyo, Japan; Tokyo Metropolitan Institute for Geriatrics and Gerontology, Itabashi, Tokyo, Japan; Tokyo Metropolitan Institute for Geriatrics and Gerontology, Itabashi, Tokyo, Japan; Tokyo Metropolitan Institute for Geriatrics and Gerontology, Itabashi, Tokyo, Japan; National Center for Geriatrics and Gerontology, Obu, Aichi, Japan; Tokyo Metropolitan Institute for Geriatrics and Gerontology, Itabashi, Tokyo, Japan; Tokyo Metropolitan Institute for Geriatrics and Gerontology, Tokyo Metropolitan Institute for Geriatrics and Gerontology, Tokyo, Japan; Tokyo Metropolitan Institute for Geriatrics and Gerontology, Itabashi, Tokyo, Japan

## Abstract

Remote interventions for prefrail older people are more feasible than for frail older people, but the benefits of using digital health technologies (DHT) have not been comprehensively investigated. This cross-sectional study examined whether DHT use in prefrail older people is associated with better physical/cognitive/mental function. Participants aged ≥70 years with prefrailty, as defined by the Japanese Cardiovascular Health Study criteria, were recruited from the Itabashi Longitudinal Study on Aging between 2022–2024. Exclusion criteria comprised impaired activities of daily living, Mini-Mental State Examination (MMSE) scores < 23, or physician-diagnosed dementia. DHT ownership and usage, such as messaging, searching, video calls, and video viewing, were self-reported. Physical characteristics included muscle strength, body mass index, and skeletal muscle mass index. Cognitive function was assessed by MMSE, and mental health was evaluated using WHO-5. Binomial logistic regression, adjusted for demographics, lifestyle, and socioeconomic status, calculated adjusted odds ratios (aORs) and 95% confidence intervals (CIs), with non-DHT ownership as the reference. Among 2,681 participants, eligible 862 (mean age 77.0±4.5 years, 44.0% women) were analyzed. DHT ownership was inversely associated with lower handgrip strength (< 28kg for men, < 18kg for women) (aOR: 0.68; 95% CI: 0.47–1.00) and lower MMSE (< 27) scores (aOR: 0.65; 95% CI: 0.44–0.98). The use of searching (aOR: 0.49; 95% CI: 0.28–0.83) and video calls (aOR: 0.58; 95% CI: 0.38–0.88) were inversely associated with lower WHO-5 scores. These findings suggest DHT may support physical/cognitive/mental health in prefrail older adults.

